# Overexpression of *StBIN2* Improves Salt Stress Tolerance in Potato

**DOI:** 10.3390/ijms27177747

**Published:** 2026-08-29

**Authors:** Shifeng Liu, Yichen Wang, Juqin Wang, Rui Peng, Chengcheng Cai, Lang Yan, Xianjun Lai

**Affiliations:** 1 Panxi Crop Improvement Key Laboratory of Sichuan Province, College of Agricultural Science, Xichang University, Liangshan 615300, China; 13838657723@163.com (S.L.); 18040471326@163.com (J.W.); por_0904@foxmail.com (R.P.); 18782236003@163.com (L.Y.); 2 School of Foreign Languages and Cultures, Xichang University, Liangshan 615300, China; 18881047880@163.com; 3 College of Agronomy, Sichuan Agriculture University, Chengdu 611130, China; caichengcheng@stu.sicau.edu.cn

**Keywords:** *Solanum tuberosum*, salt stress, *StBIN2*, antioxidant enzyme activities

## Abstract

Salt stress has become one of the major abiotic stress factors limiting sustainable crop production worldwide, and potato, as the fourth largest food crop globally, is particularly severely affected by saline and other environmental stresses in terms of its growth, development, yield, and quality. *StBIN2* belongs to the GSK3 family of proteins, and numerous studies have confirmed that GSK3 family members widely regulate diverse abiotic stress responses and developmental processes in plants. However, the specific function and underlying mechanism of *StBIN2* in the salt stress response of potato remain unclear. In this study, using the potato cultivar ‘Chuanyu 10’ as experimental material, we successfully isolated and cloned the *StBIN2* gene and systematically investigated its biological function under salt stress. Subcellular localization analysis revealed that the StBIN2 protein is localized in the nucleus. Tissue-specific expression pattern analysis showed that *StBIN2* transcript levels were significantly higher in leaves and tuber tissues than in other tissues. Under 200 mM NaCl salt stress treatment, *StBIN2*-overexpressing potato lines exhibited enhanced salt stress tolerance compared with WT plants, whereas gene-silenced lines displayed a hypersensitive phenotype to salt stress. Physiological parameter measurements demonstrated that the activities of superoxide dismutase (SOD) and catalase (CAT) in overexpressing transgenic plants were significantly upregulated relative to those in the WT, whereas the levels of malondialdehyde (MDA) and hydrogen peroxide (H_2_O_2_) were markedly reduced. Collectively, these experimental results confirm that *StBIN2* significantly enhances salt tolerance in transgenic potato, indicating that potato *StBIN2* positively participates in the physiological regulation of salt stress. This work lays an important foundation for further elucidation of the functional mechanism of *StBIN2* within the plant abiotic stress response network.

## 1. Introduction

**Figure 1:**
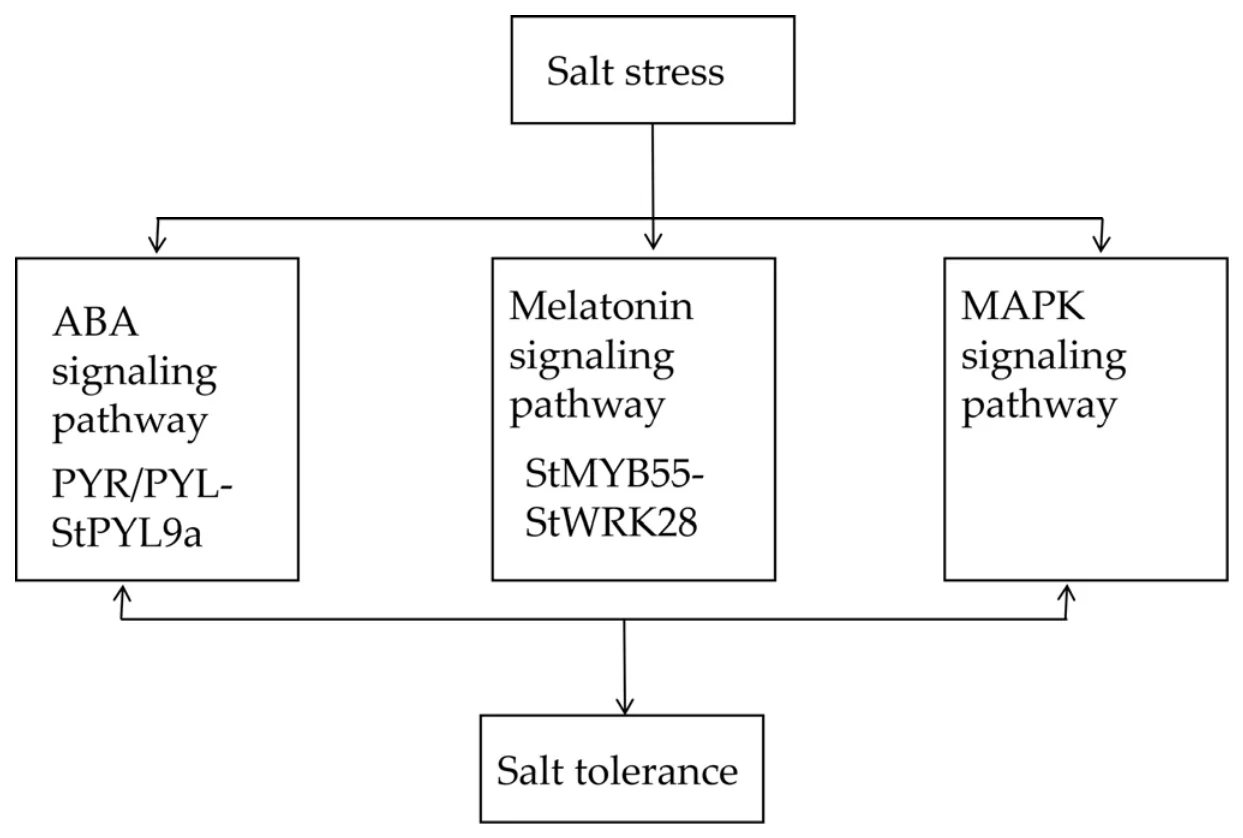
Multi-pathway regulatory network for salt tolerance in potato under salt stress.

Potato is the fourth largest food crop in the world after wheat, rice, and maize [1]. It is now widely cultivated across the globe and is one of the most important high-yield crops used for both food and vegetable purposes. Potato tubers contain large amounts of starch, which provides a rich source of energy for the human body, and are also abundant in proteins, amino acids, vitamins, and minerals [2]. In particular, their vitamin content is the most comprehensive among all food crops [3]. Soil salinization is currently one of the major and widespread challenges [4]. In 2021, the global area of salt-affected soils exceeded 833 million hectares [5]. In recent years, due to climate change and factors such as unreasonable reclamation and irrigation, the problem of land salinization has continued to intensify worldwide [6]. Salt stress has thus become one of the important environmental factors that constrain crop growth and reduce yield [7]. Potato is moderately sensitive to salt stress, which severely interferes with its metabolic activities, leading to physiological dehydration, wilting, and even death, ultimately resulting in yield reduction [8]. PYR/PYL serve as ABA receptors [9], and overexpression of *StPYL9a* markedly enhances potato growth and survival under salt stress through coordinated regulation of antioxidant enzymes and osmotic balance [10]. Melatonin, as an endogenous antioxidant and stress-signaling mediator [11], confers salt tolerance via the StMYB55-StWRKY28 module that promotes flavonoid accumulation [12]. MAPKs are extensively involved in growth, development, and abiotic stress responses [13], and overexpression of *StMAPKK5* improves tolerance to both drought and salt stress [14]. Collectively, these findings indicate that PYL, melatonin signaling, and MAPK cascades all contribute to stress resistance in potato (Figure 1). Thus, further cloning and functional dissection of stress-related genes hold promise for providing both theoretical foundations and molecular targets for genetic improvement and cultivation management, which is of substantial research and practical significance.

BIN2, as a core negative regulatory kinase in the brassinosteroid (BR) signaling pathway, plays a central regulatory role in responses to various abiotic stresses, including cold, heat, drought, and salt stress, by phosphorylating multiple downstream transcription factors such as ICE1, HsfA1d, TINY, and RD26 [15]. The dynamic changes in its kinase activity enable plants to flexibly switch between normal growth and stress defense. In *Arabidopsis thaliana*, BIN2 functions as a molecular switch for the transition to robust growth after salt stress. AtBIN2 interacts with SOS2 and phosphorylates SOS2 at the T172 site to inhibit its kinase activity, thereby regulating the rapid recovery of *Arabidopsis* after salt stress [16]. Additionally, AtHOP1 and HOP2 participate in salt tolerance by affecting the co-chaperone heat shock protein (HSP) 70-HSP90 organization and the nucleo-cytoplasmic partitioning of BIN2, thereby influencing BR signaling [17]. ABA is a key hormone in plant responses to osmotic stress, and its signaling pathway is rapidly activated upon salt stress, inducing a series of downstream defense responses such as stomatal closure and accumulation of osmotic regulatory substances [18]. BIN2, as a critical crosstalk node between ABA and BR signaling pathways, can not only regulate BR signaling but also mediate ABA signaling, thereby participating in the regulation of plant salt tolerance [19]. SnRK2s are key proteins in the ABA signaling pathway [20]. In *Arabidopsis*, AtBIN2 interacts with SnRK2.2/2.3/2.6 and enhances their kinase activity, positively regulating the ABA signaling pathway [21]. In potato, *StBIN2* interacts with StSnRK2.3 and enhances its phosphorylation activity [22]. Under cold stress, BIN2 proteins from both rubber tree and *Arabidopsis* can interact with the transcription factor ICE1, promoting ICE1 degradation and thereby enhancing cold tolerance [23]. In terms of drought stress, AtBIN2 phosphorylates and stabilizes the NAC transcription factor RD26, promoting drought stress responses [24]. In summary, BIN2 protein plays an important role in plant responses to abiotic stress; however, the specific function of *StBIN2* in salt stress tolerance in potato still requires further in-depth investigation.

In this study, to elucidate the role of *StBIN2* in salt tolerance in potato, bioinformatics approaches were employed to analyze the gene structure, promoter cis-acting elements, protein conserved domains, phylogenetic relationships, and tissue expression patterns of *StBIN2*, indicating its responsiveness to stress stimuli. To investigate the function of *StBIN2* in regulating salt tolerance in potato, we generated *StBIN2* overexpression and silencing materials. Potato seedlings were treated with 200 mM sodium chloride, and the results showed that the plant height of overexpression lines was significantly greater than that of silencing lines and WT plants. Further analysis revealed that *StBIN2* enhances antioxidant enzyme activity to reduce ROS accumulation, thereby protecting cells from oxidative damage. In summary, this study not only identifies a novel salt tolerance-related gene in potato but also provides a theoretical basis for breeding salt-tolerant potato varieties and offers reference insights for salt tolerance research in other crops.

## 2. Results

### 2.1. Cloning and Corresponding Bioinformatics Analysis of the StBIN2 Gene

**Figure 2:**
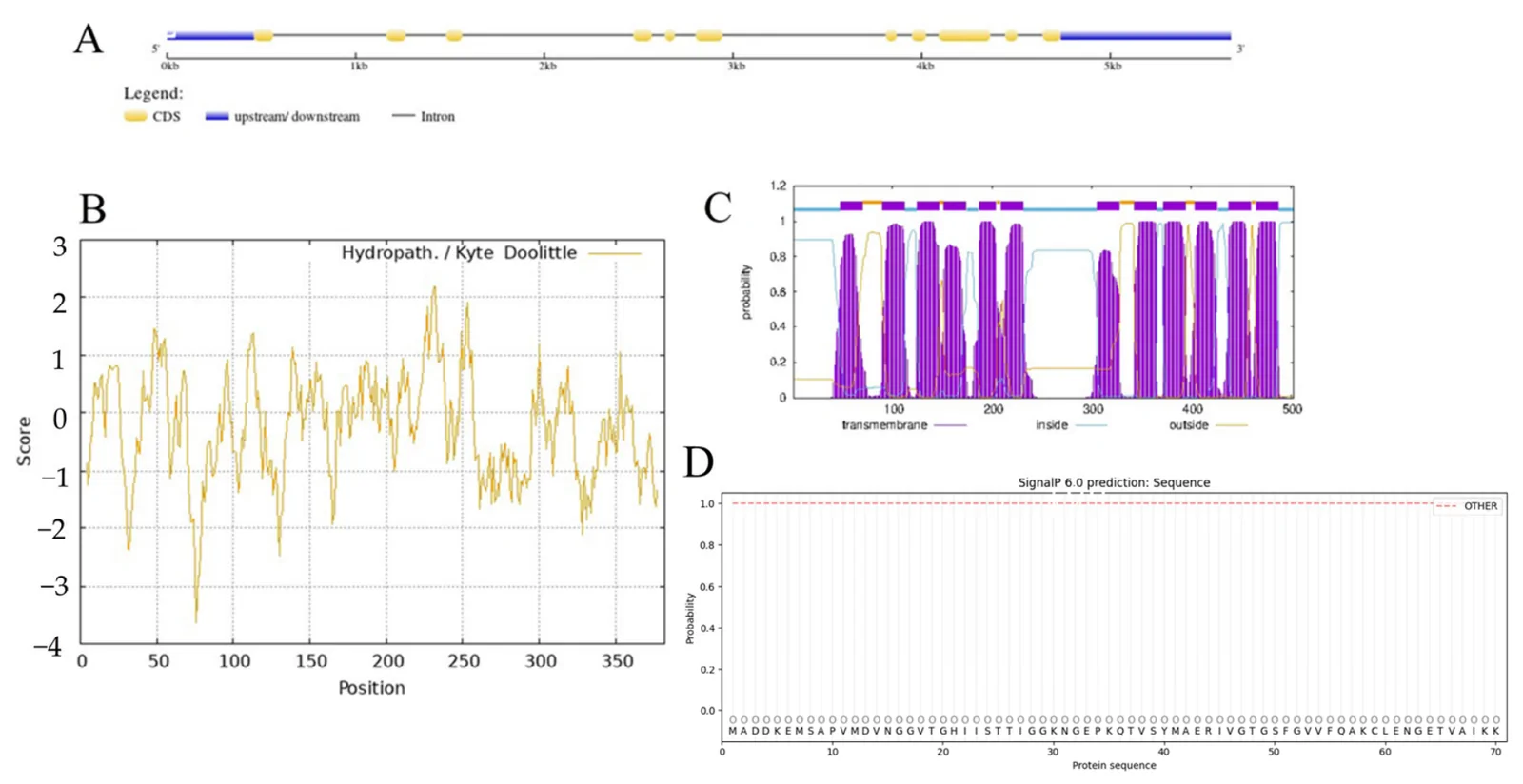
Bioinformatics analysis of *StBIN2*. Notes: (**A**) Gene structure analysis of *StBIN2*; (**B**) Kyte–Doolittle hydrophobicity/hydrophilicity analysis of the protein sequence; (**C**) Transmembrane domain prediction of the StBIN2 protein; (**D**) Signal peptide prediction of the StBIN2 protein.

*StBIN2* belongs to the GSK3 protein family. The full-length gene is 6736 base pairs in length, containing 11 introns and 10 exons, and encodes 383 amino acids (Figure 2A). Among these, serine (Ser, S) accounts for 11.9%, glutamic acid (Glu, E) accounts for 7.3%, lysine (Lys, K) accounts for 7.3%, and glycine (Gly, G) accounts for 6.6%, all of which are the most abundant amino acids. The molecular weight and isoelectric point (pI) of this protein are 43.568 kDa and 8.47, respectively. Its grand average of hydropathicity (GRAVY) is −0.727, indicating that it is a hydrophobic protein (Figure 2B). The instability index of StBIN2 protein is 41.47, slightly higher than the universal threshold of 40.0, indicating that the protein is unstable. Prediction of its transmembrane domains and signal peptide revealed that the StBIN2 protein has no transmembrane domains or signal peptide (Figure 2C,D). The subcellular localization prediction of StBIN2 protein was performed using Cello online tool, and the results showed that StBIN2 protein is located in the cytoplasm and nucleus. The secondary structure of *StBIN2* was predicted using PSIPRED. From the prediction results, it can be seen that random coils are the most abundant structural elements in the StBIN2 protein, followed by α-helices, while β-sheets are the least abundant (Supplementary Figure S1). This distribution pattern of secondary structures may play an important role in the function of these proteins. Subsequently, based on the secondary structure, we predicted the tertiary structure of *StBIN2*; the results showed that the per-residue confidence score (pLDDT) calculated by AlphaFold for *StBIN2* is 89.56, indicating that this tertiary structure can be applied to experimental research to a certain extent.

**Figure 3:**
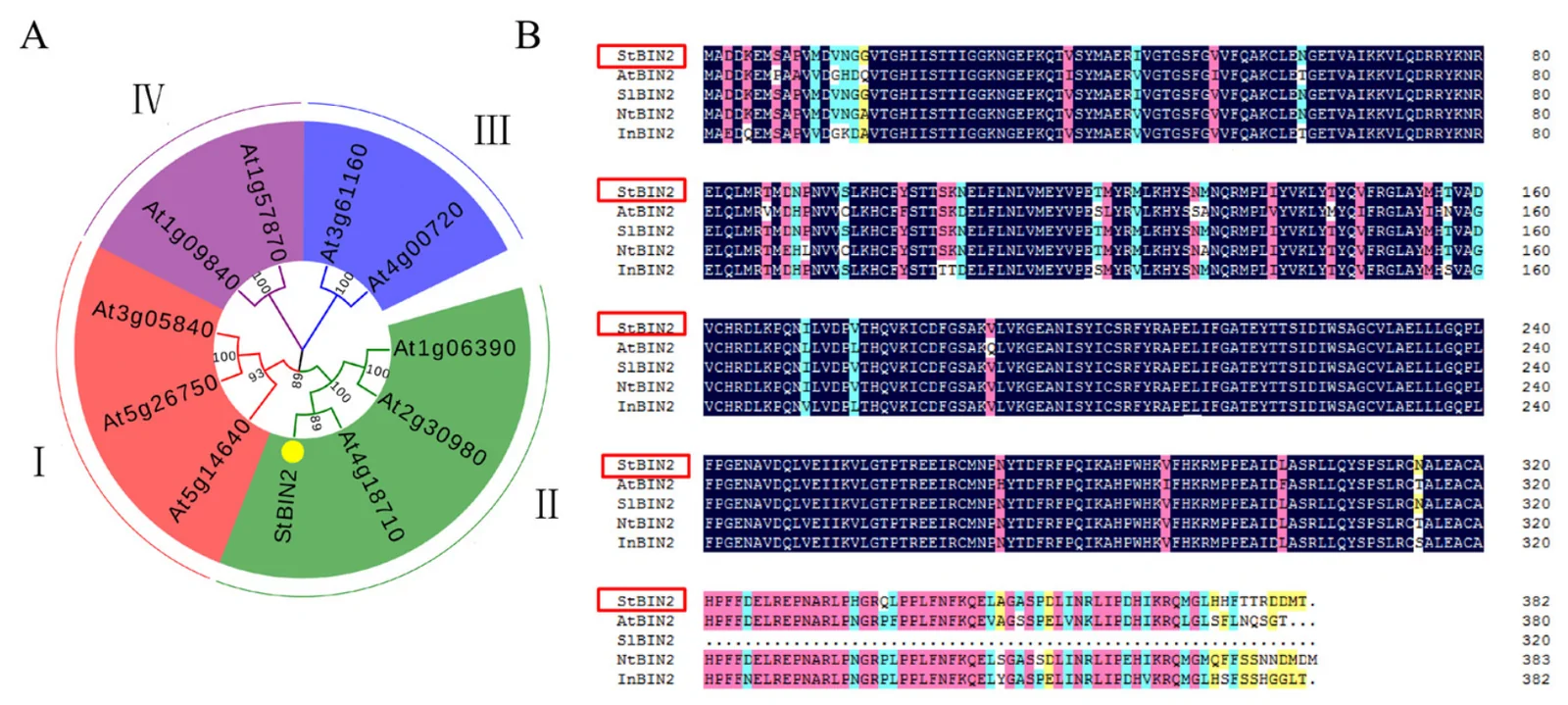
Sequence alignment and phylogenetic tree analysis of the StBIN2 protein. Notes: (**A**) Phylogenetic tree analysis of Arabidopsis GSK3 and *StBIN2*. *StBIN2* is indicated by a yellow circle. The full-length amino acid sequences of *Arabidopsis* were obtained from the public TAIR database. (**B**) Amino acid sequence alignment of BIN2 from *Arabidopsis,* potato, tomato, tobacco, and morning glory. The red box indicates the StBIN2 protein.

The GSK3 proteins in *Arabidopsis thaliana* are among the earliest studied and most functionally characterized members of this family. To investigate the function of *StBIN2*, a phylogenetic analysis was performed between potato *StBIN2* and the GSK3 protein family of *Arabidopsis* (the *Arabidopsis* GSK3 sequences are listed in Supplementary S1). The *Arabidopsis* GSK3 proteins are divided into four classes, and potato *StBIN2* was classified into the second class. *StBIN2* showed the highest homology with the *Arabidopsis* At4g18710 (AtBIN2) protein (Figure 3A). In *Arabidopsis*, AtBIN2 interacts with the SOS2 protein to mediate the SOS (Salt Overly Sensitive) signaling pathway, and through phosphorylation, it activates SOS1 to expel excess intracellular Na^+^, thereby maintaining ion homeostasis and enhancing plant salt tolerance [16]. This suggests that *StBIN2* may similarly enhance salt tolerance in plants. Subsequently, the amino acid sequence of potato BIN2 was aligned and analyzed with BIN2 sequences from tomato, tobacco, morning glory (Ipomoea), and *Arabidopsis*. The results revealed that BIN2 proteins from different species all possess a conserved serine/threonine kinase domain (Figure 3B). This finding further confirms that BIN2 belongs to the glycogen synthase kinase GSK3 family and is a highly conserved class of protein kinases in plants.

### 2.2. Expression Pattern Analysis of StBIN2

**Figure 4:**
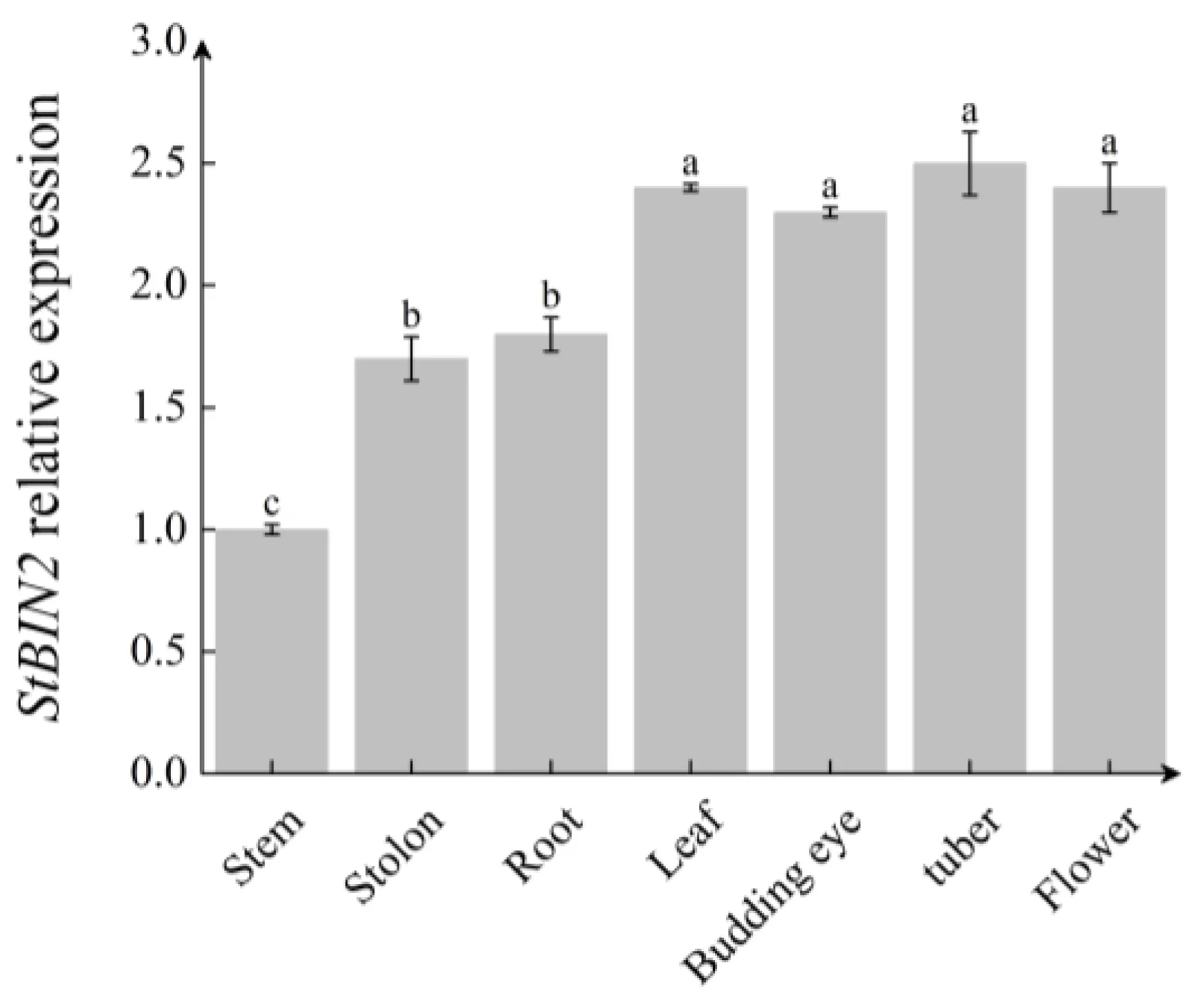
Tissue-specific expression analysis of *StBIN2*. Data are the means ± SD of three biological replicates. Different small letters represent significant differences (*p* < 0.05).

The tissue-specific expression of a gene can, to some extent, indicate its function [25]. In this study, qPCR technology was used to measure the expression levels of *StBIN2* in potato roots, stems, leaves, flowers, buds, tubers, and bud eyes. The results showed that *StBIN2* was highly expressed in flowers, tubers, bud eyes, and leaves, with moderate expression also detected in roots and stems (Figure 4). In addition, by retrieving the Fragments Per Kilobase of transcript per Million mapped reads (FPKM) values of the *StBIN2* gene from the Spud DB database, the expression trend was found to be largely consistent with the qPCR results, further supporting the hypothesis that this gene plays important functions in above-ground organs and tubers.

### 2.3. Subcellular Localization of StBIN2

**Figure 5:**
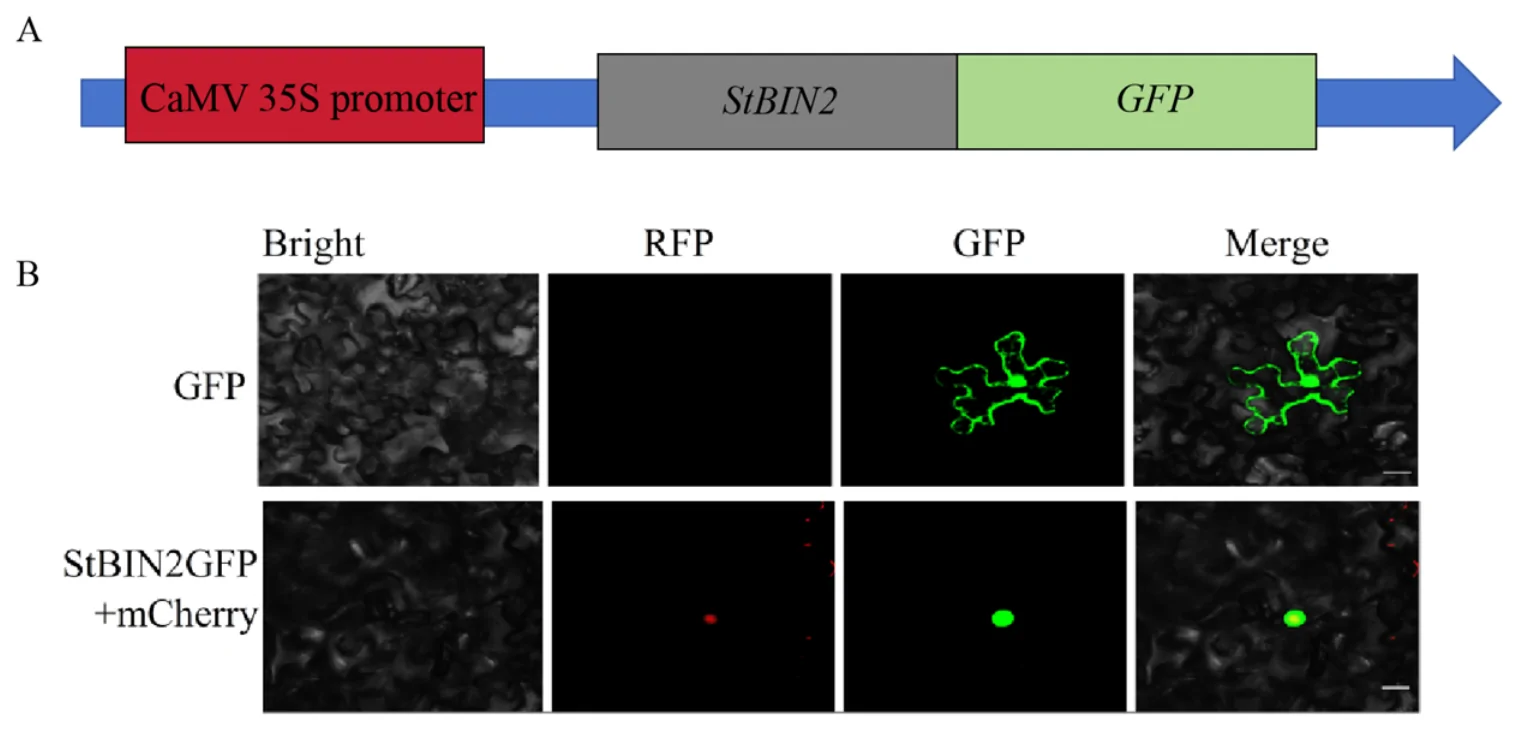
Subcellular localization of *StBIN2* in tobacco. Note: (**A**) *StBIN2-GFP* gene structure. (**B**) Preliminary localization of StBIN2 protein in tobacco. Scale bar = 20 μm.

Biological cells possess highly complex subcellular structures, and the correct localization of proteins within tissues and cells is crucial for their biological functions as well as for the normal performance of cellular functions [26]. To investigate the subcellular localization of the StBIN2 protein, the researchers cloned its coding sequence (CDS) and constructed an expression vector for the *StBIN2-GFP* fusion protein driven by the 35S promoter (Figure 5A). This construct was transformed into *Agrobacterium*, cultured, incubated, and then infiltrated into tobacco plants. Fluorescence signals were finally detected using laser scanning confocal microscopy, and the subcellular localization of the protein was preliminarily determined. As shown in Figure 5B, the fluorescence signals of the 35S::StBIN2-GFP construct were all observed on the cell nucleus. The prediction results were highly consistent with the subcellular localization results, thereby confirming that the *StBIN2* gene encodes a protein localized to the nucleus.

### 2.4. StBIN2 Promoter Analysis

**Figure 6:**
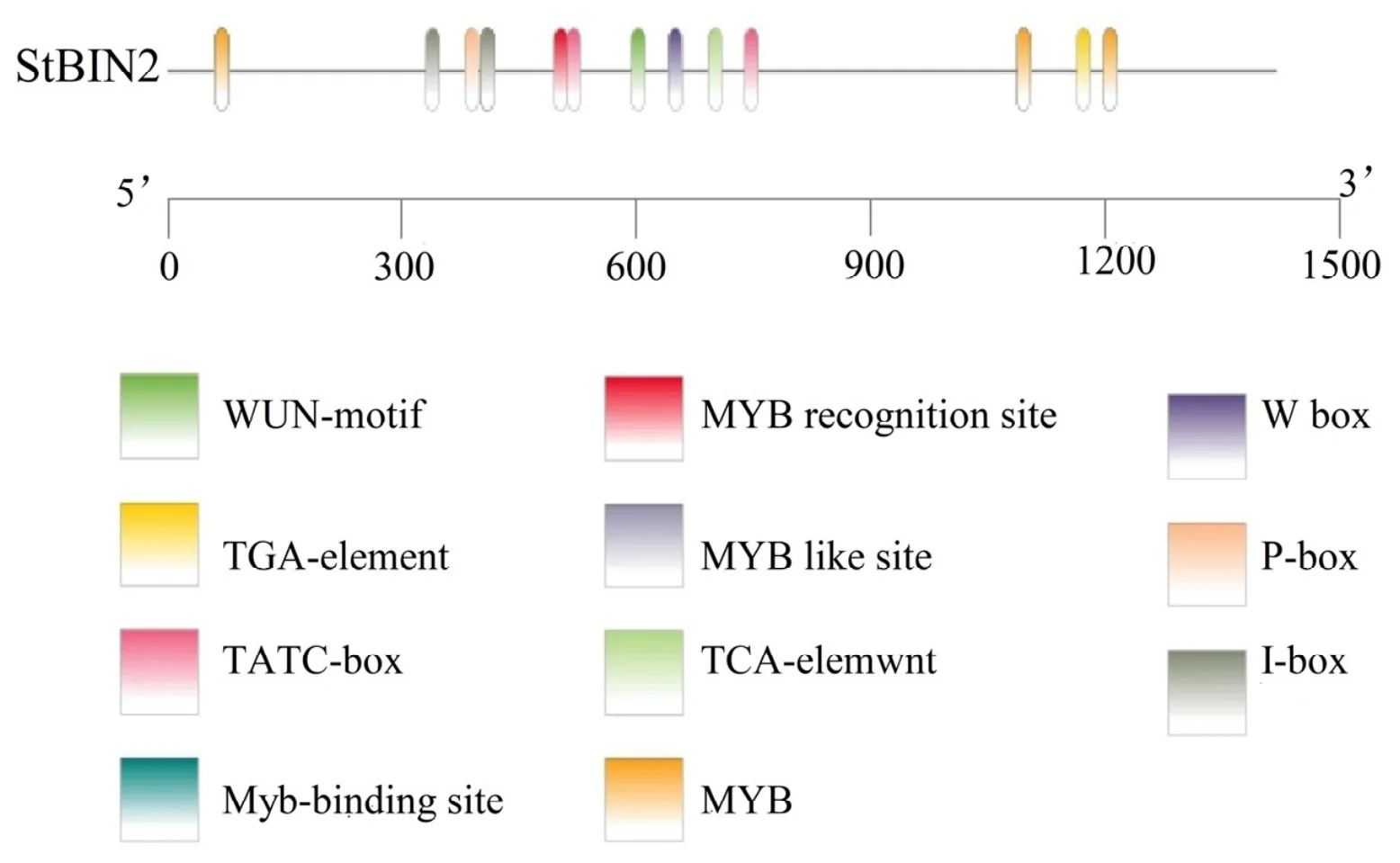
Cis-acting Element Analysis of the *StBIN2* Promoter.

The expression of eukaryotic genes is typically subject to precise regulation by upstream regulatory proteins, such as transcription factors [27]. To investigate the regulatory elements of *StBIN2*, this study analyzed the 1500 bp promoter region upstream of the gene (Figure 6; Table 1). Multiple hormone-responsive and stress-responsive cis-regulatory elements (CREs) were identified, including the ABA-responsive elements ABRE, ABRE3a, and ABRE4, the gibberellin-responsive element P-box, and the stress-responsive element TC. These results indicate that the expression of *StBIN2* is regulated by ABA and GA signaling pathways, suggesting that this gene may be involved in hormone-responsive regulation during potato growth and development.

**Figure 7:**
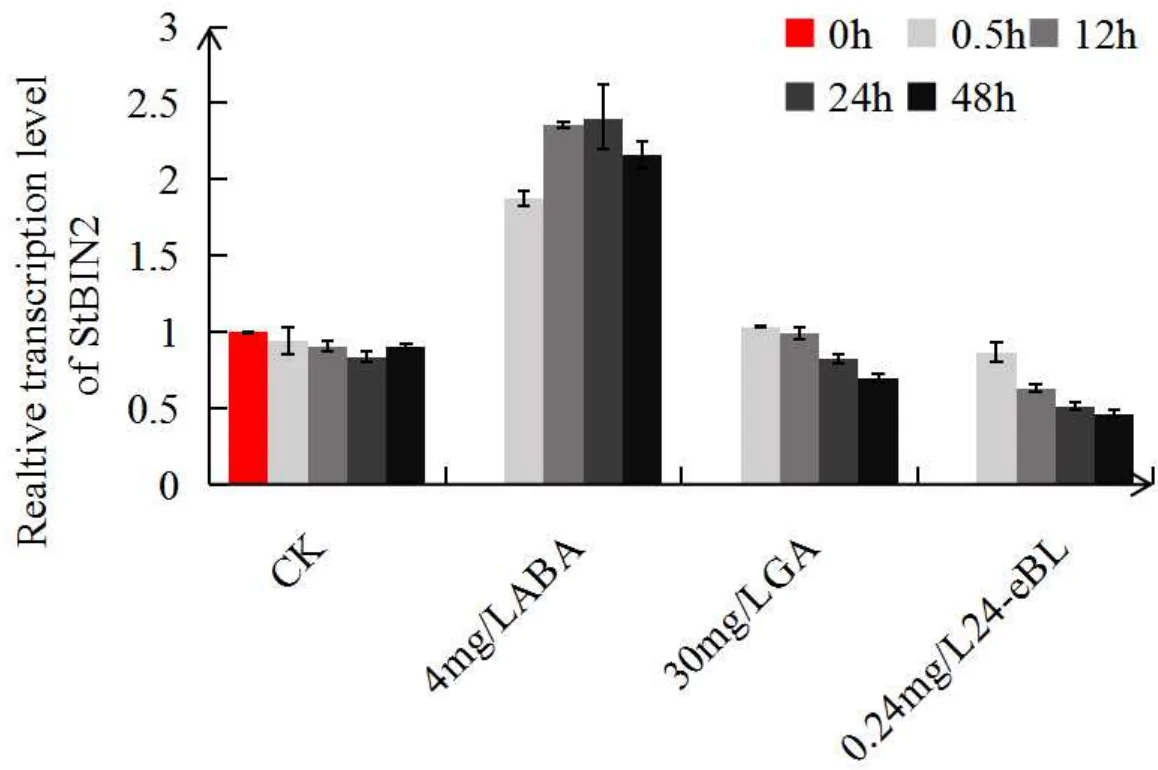
Expression of *StBIN2* gene under three hormone treatments.

To verify whether exogenous ABA, GA, and BR could affect the changes in *StBIN2* gene expression in potato tubers, we selected post-harvest potato tubers of uniform size. After wound healing treatment, the tubers were soaked separately in 4 mg/L ABA, 30 mg/L GA, and 0.24 mg/L 24-epibrassinolide (24-eBL), with distilled water used as the control (CK). After soaking for 30 min, the tubers were air-dried. Using the apical bud eye as the center, samples of the bud eyes were collected with a cylindrical punch (3 mm in diameter, 5 mm in height). RNA was extracted from the bud eyes at 0 h, 0.5 h, 12 h, 24 h, and 48 h, and the transcriptional level of the *StBIN2* gene was subsequently determined. The qPCR results showed that the water treatment had no significant effect on *StBIN2* gene expression. After GA and BR treatments, the expression levels of *StBIN2* were 1.03-, 0.98-, 0.82-, and 0.69-fold and 0.86-, 0.62-, 0.51-, and 0.46-fold of the 0 h level, respectively. The decline of *StBIN2* expression under BR treatment was faster than under GA treatment, indicating that BR had a stronger inhibitory effect on *StBIN2* than GA. On the other hand, under ABA treatment, *StBIN2* gene expression first increased and then decreased, reaching 1.87-, 2.35-, 2.39-, and 2.16-fold of the 0 h level, indicating that *StBIN2* responds to ABA induction (Figure 7). In summary, among the three hormone treatments, ABA had the greatest effect on *StBIN2* gene expression, followed by BR, while GA had the least effect, suggesting that *StBIN2* may maintain its function through ABA signaling.

### 2.5. Overexpression of StBIN2 Enhances Salt Tolerance in Potato

As a bridge between ABA and BR hormone signal transduction, BIN2 has been shown to play positive roles in various abiotic stresses. By retrieving the FPKM values of the *StBIN2* gene from the Spud DB database, the study found that under drought, cold stress, and high-salt treatments, the expression of *StBIN2* was significantly upregulated (Supplementary Table S2), indicating that *StBIN2* may serve as a key node in the crosstalk between ABA and BR signaling, positively regulating potato defense responses to multiple abiotic stresses, including drought, low temperature, and high salinity.

**Figure 8:**
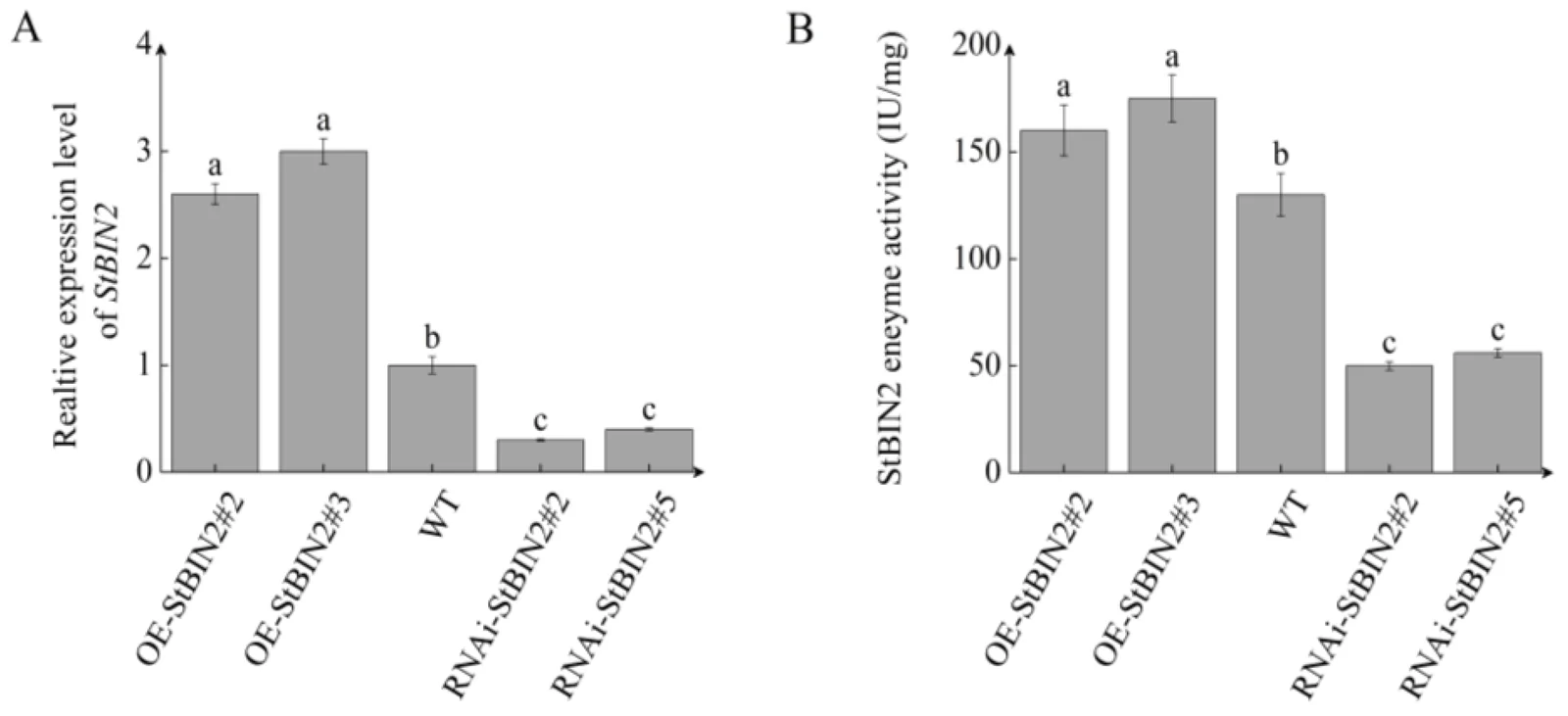
Expression levels and enzymatic activities of *StBIN2* in different transgenic lines. Note: (**A**) Expression levels of *StBIN2* in different transgenic materials. (**B**) Enzyme activity of *StBIN2* in different genetically modified materials. Data are the means ± SD of three biological replicates. Different small letters represent significant differences (*p* < 0.05).

To elucidate the function of *StBIN2* in transgenic potato in response to salt stress, we constructed different *StBIN2* expression vectors. Through *Agrobacterium*-mediated transformation of stem segments, a total of 23 RNAi lines and 27 overexpression (OE) lines were obtained. RNAi lines 2 and 5, and OE lines 2 and 3 were selected for further analysis by qRT-PCR (Figure 8A). Subsequently, the BIN2 kinase activity in the relevant lines was measured using a kinase activity assay kit (Figure 8B). The results were consistent with the qPCR data, showing that *StBIN2* kinase activity in the overexpression lines was significantly higher than that in the RNAi and WT materials.

**Figure 9:**
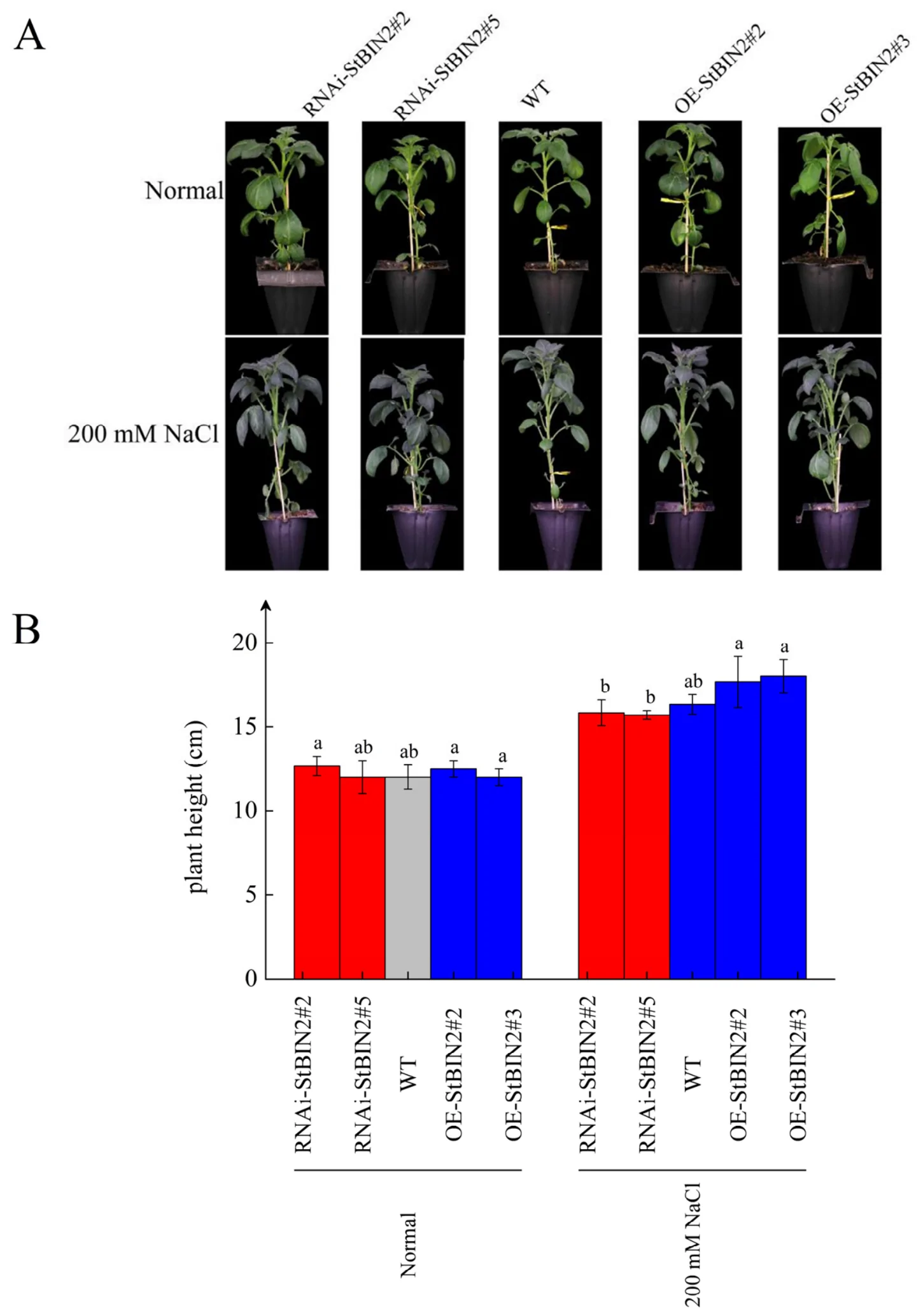
Growth phenotypes of different lines under salt stress. (**A**) Phenotypes of different genetically modified materials under salt stress. (**B**) Plant height of different transgenic materials under salt stress. Data are the means ± SD of three biological replicates. Different small letters represent significant differences (*p* < 0.05).

To explore the contribution of *StBIN2* to salt tolerance, we cultured the transgenic potato plantlets under normal conditions for approximately 3 weeks, and then added 200 mM sodium chloride to the soil matrix to impose salt stress. Under normal growth conditions, no significant phenotypic differences were observed between the transgenic lines and the wild-type lines. However, after 10 days of salt stress treatment, phenotypic observation revealed that all potato plants (including both transgenic and WT lines) suffered damage. Plant height measurements showed that, compared with the wild type, *OE-StBIN2#2* and *OE-StBIN2#3* exhibited increases of 1.13-fold and 1.12-fold, respectively, while the two RNAi lines showed reductions of 0.96-fold (Figure 9A,B). Leaf wilting was relatively mild in the overexpression lines, whereas the RNAi lines displayed the most severe wilting. These results indicate that overexpression of *StBIN2* can enhance potato tolerance to salt stress.

**Figure 10:**
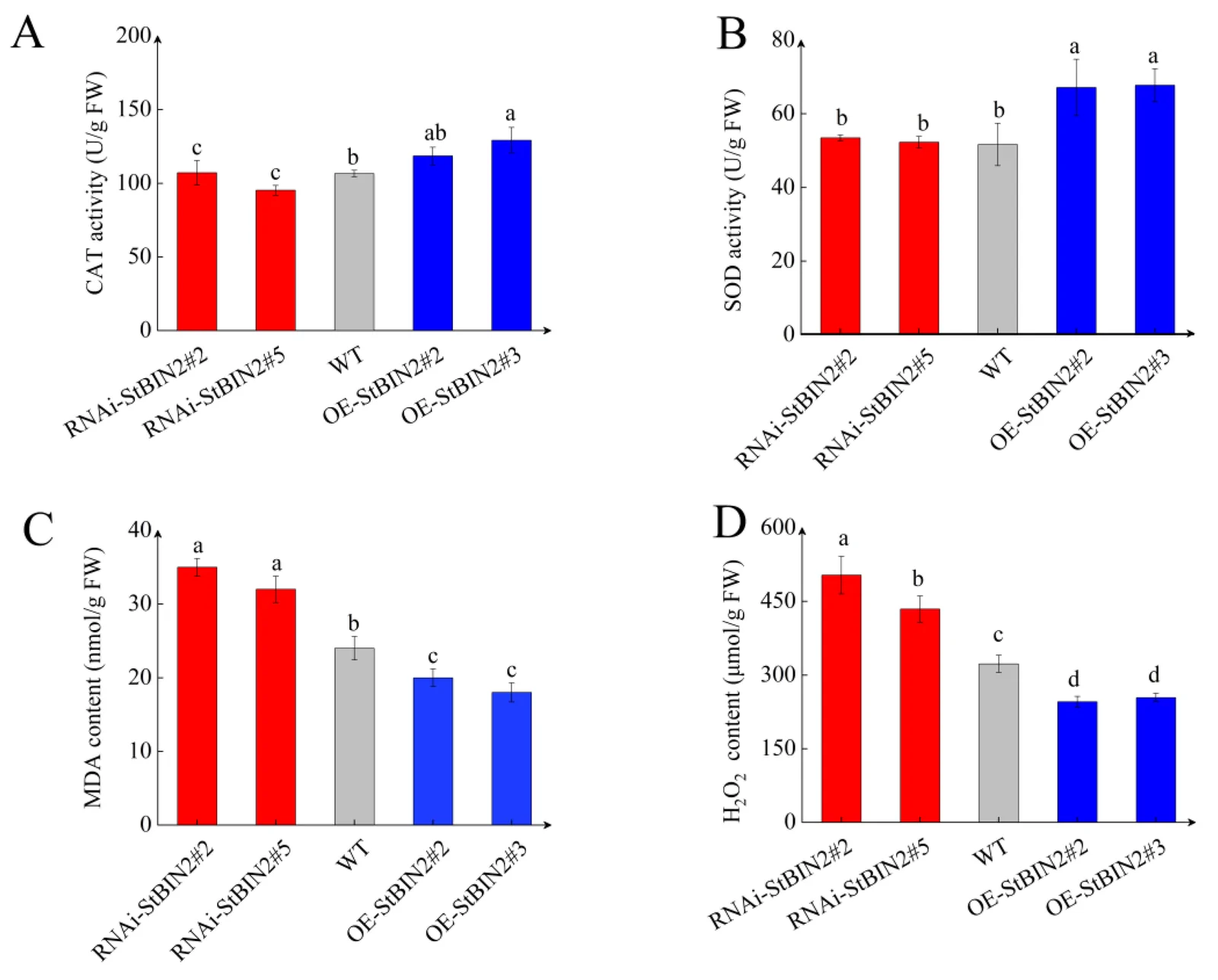
Overexpression of *StBIN2* enhances antioxidant enzyme activity under salt stress. (**A**) CAT enzyme activity of different transgenic materials under salt stress. (**B**) SOD enzyme activity of different transgenic materials under salt stress. (**C**) MDA content of different transgenic materials under salt stress. (**D**) H_2_O_2_ content of different transgenic materials under salt stress. Data are the means ± SD of three biological replicates. Different small letters represent significant differences (*p* < 0.05).

When plants are subjected to stress, they often enhance the activity of peroxidase enzymes to scavenge excess reactive oxygen species (ROS), thereby increasing their tolerance to stress [28]. Under 200 mM salt stress, the CAT and SOD enzyme activities in the overexpression lines were significantly higher than those in the silenced lines and the wild type (Figure 10A,B)., whereas the contents of MDA and H_2_O_2_ showed the opposite trend (Figure 10C,D). These results indicate that *StBIN2* can enhance potato tolerance to salt stress by increasing the activities of antioxidant enzymes (SOD and CAT).

## 3. Discussion

Soil salinization is one of the core abiotic stress challenges facing global agricultural production. High concentrations of soluble salts disrupt the soil water potential balance, triggering osmotic stress and excessive accumulation of reactive oxygen species (ROS) in plant cells, ultimately leading to physiological metabolic disorders and inhibited growth and development, seriously threatening crop yield and food security [29]. As an important food crop, potato is widely favored by people worldwide. Salt stress is one of the major abiotic stresses threatening potato growth and yield, and in severe cases, it can cause up to 60% yield loss [30]. GSK3 family proteins play important “signal hub” roles in plants, being widely involved in growth and development, hormone signal transduction, and responses to various environmental stresses, including salt stress [31]. As a key negative regulator of the brassinosteroid (BR) signaling pathway, the function of BIN2 protein has been relatively well characterized. Under high-temperature stress conditions, the BIN2 protein in *Arabidopsis* interacts with the class A1 heat shock transcription factors (HsfA1s), the primary heat-responsive transcription factors, thereby enhancing the thermotolerance of *Arabidopsis* [32]. Cell replication is the basis for organismal growth, development, and damage repair. Recent studies have found that when plants encounter replication stress, BIN2 kinase is activated and phosphorylates the SOG1 protein, preventing its ubiquitination and degradation, thereby activating the DNA damage repair response [33]. Although the functions of BIN2 in enhancing abiotic stress tolerance have been documented in other crops, research on the salt stress response mechanism of potato BIN2 remains relatively scarce.

In this study, the GSK3 family protein StBIN2 was amplified from the potato cultivar ‘Chuanyu 10’. Gene structure and phylogenetic analysis revealed that its full-length sequence is 1149 base pairs (bp) in length, encoding 382 amino acids. Protein property analysis showed that the theoretical molecular weight of the StBIN2 protein is 43.568 kDa, the theoretical isoelectric point is 8.47, and the grand average of hydropathicity (GRAVY) is 0.727, classifying it as a hydrophobic protein; its instability index is 41.47, categorizing it as an unstable protein. Phylogenetic tree analysis of the StBIN2 protein using MEGA7.0 software revealed that this protein shares conserved nucleotide homology with the *Arabidopsis* AtBIN2 protein (Figure 3A) and exhibits the closest genetic relationship.

Subcellular localization of proteins plays an important role in regulating their activity or function, and investigating the subcellular localization of the BIN2 protein is helpful for elucidating its function [34]. As a core negative regulatory kinase in BR signal transduction, BIN2’s nucleocytoplasmic distribution dynamically shifts in response to BR signal intensity and external stress, thereby precisely regulating the localization and activity of downstream transcription factors to achieve a balance between plant growth and stress resistance. Therefore, studying the subcellular localization of BIN2 contributes to understanding its functional regulatory mechanisms. To accurately determine the intracellular localization of the StBIN2 protein, identify its functional domains, and gain deeper insight into its mechanism of action, we introduced the transient expression fusion vector *StBIN2-pCAMBIA2300-GFP* into *Nicotiana benthamiana* leaf epidermal cells using *Agrobacterium*-mediated transient transformation. The results revealed its nuclear localization (Figure 5B), consistent with the predicted results.

Gene expression profiles are generally closely related to gene function [35]. *NjBIN2* is an important member of the GSK3 family in *Nardostachys jatamansi*. RT-qPCR results showed that the expression level of *NjBIN2* was highest in roots, while relatively lower in leaves, flowers, and stems, indicating that the *NjBIN2* gene is mainly expressed in roots [36]. Multiple studies have shown that the *StBIN2* gene exhibits tissue-specific expression throughout development, as reflected in the differential expression levels among different tissues and organs. In this experiment, *StBIN2* showed higher expression levels in flowers, tubers, bud eyes, and leaves (Figure 4), suggesting that this gene has higher sensitivity to abiotic stress in newly developing organs, while its transcript levels were lower in roots and stems.

As a key negative regulatory kinase in the BR signaling pathway, the promoter region of *StBIN2* is enriched with various hormone- and stress-responsive cis-regulatory elements (CREs), including ABA-responsive elements (ABRE, ABRE3a, ABRE4) [37], the gibberellin-responsive element P-box [38], and the stress-responsive element TC [39], suggesting that its expression is coordinately regulated by ABA and GA signals. To verify this hypothesis, we treated potato tubers with exogenous hormones. The results showed that ABA significantly induced *StBIN2* expression, while BR and GA exerted inhibitory effects (Figure 7). This antagonistic regulatory pattern between ABA and BR/GA is highly consistent with its functional roles in growth inhibition and stress adaptation, suggesting that *StBIN2* may participate in regulating the physiological transition of potato tubers between normal growth and development and stress responses by integrating mutually antagonistic hormone signals.

The most direct strategy in gene function research is to construct overexpression or silencing transgenic materials, followed by systematic evaluation of the biological functions of the target gene at the phenotypic, physiological, and molecular levels [40]. We obtained *StBIN2* overexpression and silencing materials through *Agrobacterium*-mediated transformation of stem segments. Salt stress treatment revealed that the plant height and growth vigor of the overexpression materials were superior to those of WT and silencing materials, indicating that overexpression of *StBIN2* significantly enhanced salt tolerance in potato (Figure 9A).

When plants are subjected to stress, they produce large amounts of reactive oxygen species (ROS), which can cause oxidative damage to biological macromolecules such as proteins, nucleic acids, and membrane lipids, disrupt enzyme activities, induce DNA mutations, and destabilize membrane systems, and in severe cases, even trigger programmed cell death [41]. Stress-induced hydrogen peroxide (H_2_O_2_) serves dual functions of toxicity and signaling [42]. It can directly cause oxidative cellular damage while also acting as a signaling molecule to activate the expression of antioxidant enzyme genes such as Superoxide dismutase (SOD), Catalase (CAT), Peroxidase (POD), Ascorbate peroxidase (APX), Glutathione peroxidase (GPX), and Glutathione reductase (GR), thereby increasing enzyme activities to scavenge excess Reactive Oxygen Species (ROS). The defense system composed of the aforementioned antioxidant enzymes can maintain ROS levels within a safe range, thereby alleviating cellular damage and maintaining redox homeostasis [43]. Comparative analysis in this study showed that potato seedlings overexpressing *StBIN2* exhibited stronger salt tolerance compared to the control group, with higher SOD and CAT enzyme activities and lower H_2_O_2_ contents (Figure 10A,B,D). Malondialdehyde (MDA) can serve as an indirect indicator for predicting plant stress tolerance [44] (Figure 10C). Overexpression of *StBIN2* resulted in reduced membrane damage under stress conditions, as indicated by lower MDA content compared to the control group. In summary, overexpression of *StBIN2* significantly enhanced the salt tolerance of potato seedlings by increasing antioxidant enzyme activities, reducing ROS accumulation, and alleviating membrane lipid peroxidation damage. As a key component of the BR pathway under multi-hormonal signal regulation, *StBIN2* plays an important role in coordinating plant growth and stress adaptation.

## 4. Materials and Methods

### 4.1. Gene and Protein Structure Analysis

The gene structure analysis of *StBIN2* was performed using the online tool Gene Structure Display Server (GSDS) 2.0 (https://gsds.gao-lab.org/Gsds_help.php, accessed on 6 May 2025). The physicochemical properties of the protein were predicted using the Protein Parameter (ProtParam) tool (https://web.expasy.org/protparam/, accessed on 6 May 2025), and hydrophilicity analysis was conducted through the ExPASy platform (https://www.expasy.org/, accessed on 6 May 2025). Protein transmembrane domains were predicted using TMHMM 2.0 (https://services.healthtech.dtu.dk/services/TMHMM-2.0/, accessed on 7 May 2025), and signal peptides were predicted using SignalP-5.0 (https://services.healthtech.dtu.dk/services/SignalP-5.0/, accessed on 8 May 2025). The BLASTP tool on the NCBI website (https://www.ncbi.nlm.nih.gov/, accessed on 8 May 2025) was used to perform similarity searches for the StBIN2 protein sequence, and protein sequences from other species with high similarity to the StBIN2 protein sequence were screened. Subsequently, MEGA 11.0 software was used to perform multiple sequence alignment (ClustalW) of these protein sequences, with parameters set to Poisson correction and pairwise deletion, and 1000 bootstrap replicates were performed to assess the stability of the tree. Subcellular localization of the protein was predicted using the CELLO tool (https://cello.life.nctu.edu.tw/cgi/main.cgi, accessed on 8 May 2025). Promoter cis-acting elements were analyzed on the Plant Care website (https://bioinformatics.psb.ugent.be/webtools/plantcare/html/, accessed on 8 May 2025) [45].

### 4.2. Growth Conditions and Plant Material Treatment

The transgenic *StBIN2* materials *OE-StBIN2#2*, *OE-StBIN2#3*, *RNAi-StBIN2#2*, *RNAi-StBIN2#5*, and the WT material ‘Chuanyu 10’ were maintained at Xichang University [46]. After the sterile seedlings of the transgenic materials had grown to 6–8 cm in tissue culture bottles, they were transferred to small pots containing coconut coir substrate and cultured for approximately 20 days under conditions of 22 ± 1 °C, light intensity of 2000 Lx, and a photoperiod of 16 h light/8 h dark. Subsequently, potato seedlings with essentially uniform growth status were selected for sodium chloride treatment. The seedlings were irrigated with 20 mL of 200 mmol/L sodium chloride solution. After 10 days of stress treatment, functional leaves of the potato plants were collected and frozen in liquid nitrogen.

### 4.3. Subcellular Localization

The subcellular localization experiment was performed using the *pCAMBIA2300-EGFP* vector. First, the vector was linearized with the restriction endonucleases BamHI and XbaI. The *StBIN2* gene was then cloned into the linearized vector to construct a fusion expression vector. Both the recombinant plasmid and *pBI121-NLS-mCherry* (a plant nuclear localization marker) were separately transformed into *Agrobacterium* tumefaciens strain GV3101. Positive clones were selected, and the two *Agrobacterium* strains were cultured and mixed uniformly. *Nicotiana benthamiana* leaves at 3–4 weeks of age, with flat and uniform thickness, were selected for *Agrobacterium*-mediated infiltration. After incubation at room temperature for 2–4 days, small leaf segments (approximately 0.5 cm × 0.5 cm) from the infiltrated areas were excised, and EGFP signals were observed and captured using a Nikon C2-ER confocal microscope (Nikon, Tokyo, Japan) to determine the subcellular localization of the StBIN2-EGFP fusion protein [47].

### 4.4. Tissue Expression Analysis

Tissue culture plantlets of ‘Chuanyu 10’ were transplanted into substrate in a greenhouse and grown for approximately 85 days. Roots, stems, leaves, bud eyes, tuber skins, and tuber flesh were sampled, immediately frozen in liquid nitrogen, and stored at −80 °C for subsequent RNA extraction. Total RNA was extracted from potato tissues of various organs using the SteadyPure Universal RNA Extraction Kit (AG, Changsha, China). RNA integrity was assessed on 1% (*w*/*v*) agarose gels, and RNA concentrations were determined spectrophotometrically using a NanoDrop One instrument (Thermo Fisher Scientific, Waltham, MA, USA). Aliquots of 1 μg of total RNA were reverse-transcribed into cDNA with the Evo M-MLV Reverse Transcription Kit (AG, Changsha, China) as per the supplier’s recommendations, and the resulting cDNA was employed for quantitative gene expression analyses. Potato EF1α (Elongation Factor 1-alpha) was selected as the internal reference gene, and the specific primer sequences are listed in Supplementary Table S1. The qPCR reaction system (10 µL) consisted of: 3.0 µL RNase-Free ddH_2_O, 5.0 µL 2× SYBR Mixture (Full gold, Beijing, China), 0.5 µL Primer-F, 0.5 µL Primer-R, and 1.0 µL cDNA. Amplification reactions were performed on a Bio-Rad CFX Connect real-time PCR system with the following procedure: initial denaturation at 95 °C for 20 s; 40 cycles of 95 °C for 3 s and annealing/extension at 55 °C for 30 s; followed by melt curve analysis: 95 °C for 10 s, 65 °C for 5 s, and 95 °C for 5 s. Relative expression levels were calculated using the 2^−ΔΔCt^ method, with the ΔCt value of the root sample used as the calibrator. Data were exported and processed using a 7500 Real-Time PCR System [48].

### 4.5. Exogenous Hormone Treatment

First, potato tubers of uniform size were chosen after harvest. After wound healing treatment, the tubers were soaked separately in 4 mg/L ABA, 30 mg/L GA, and 0.24 mg/L 24-epibrassinolide (24-eBL), with distilled water used as the control (CK). After soaking for 30 min, the tubers were air-dried. Using the apical bud eye as the center, samples of the bud eyes were collected with a cylindrical punch (3 mm in diameter, 5 mm in height). RNA was extracted from the bud eyes at 0 h, 0.5 h, 12 h, 24 h, and 48 h, and the transcriptional level of the *StBIN2* gene was subsequently determined.

### 4.6. Kinase Activity Assay

The activity of *StBIN2* in potato was determined using a plant BIN2 ELISA kit (Kexing, Shanghai, China). First, 100 mg of potato leaf tissue was homogenized using a homogenizer to prepare samples. Then, 40 µL of dilution buffer and 10 µL of sample were added to each well, followed by the addition of 100 µL of HRP-labeled conjugate to the sample wells. The microplate was incubated at 37 °C for 60 min. The reaction solution in each well was discarded, and the wells were washed five times with wash buffer. Then, 50 µL each of chromogenic solutions A and B were added to each well, and the microplate was incubated at 37 °C in the dark for 15 min. Finally, 50 µL of stop solution was added, and the absorbance was read at 450 nm using a microplate reader (Thermo Fisher Scientific, Waltham, MA, USA). Each 100 mg sample contained three potato leaf tissue replicates, and the experiment was performed with three biological replicates [22].

### 4.7. Physiological Index Determination

All samples were prepared by homogenizing 0.1 g of leaves in 0.01 mM phosphate buffer (pH 7.2) for physiological index determination. The homogenate was centrifuged at 12,000 rpm for 10 min at 4 °C. SOD and CAT activities were determined using SOD assay kit (Catalog No. G0101W) and CAT assay kit (Catalog No. G0105W), respectively, produced by Grace Biotechnology Co., Ltd.(Grace, Suzhou, China) MDA (Catalog No. G0109W) and H_2_O_2_ (Catalog No. G0112W) kits were used to determine MDA and H_2_O_2_ contents. All determinations were performed according to the manufacturer’s instructions (Grace, Suzhou, China) [49,50,51].

### 4.8. Data Analysis

All experiments were carried out in triplicate, and the resulting data were expressed as mean ± standard deviation (n = 3). Statistical analysis and graphical plotting were performed using SPSS 24.0 and Origin 2021 software. Student’s *t*-test was employed to evaluate significant differences, and lowercase letters above the bars in the figures denote statistically significant differences at thresholds of *p* ≤ 0.05.

## 5. Conclusions

In conclusion, the *StBIN2* gene from the potato cultivar ‘Chuanyu 10’ was cloned in the present study. Its full-length sequence is 6736 bp, with a complete coding sequence (CDS) of 1149 bp, encoding 383 amino acids. *StBIN2* was expressed in roots, stems, leaves, tubers, and tuber buds, with higher expression levels in leaves, tubers, and stolons. Subcellular localization analysis indicated that the protein is localized in the nucleus. Promoter cis-acting element analysis revealed that the promoter region of the *StBIN2* gene is enriched in multiple hormone- and stress-responsive elements. Furthermore, exogenous hormone treatment experiments confirmed that the expression of this gene is significantly induced by ABA, GA, and BR. Overexpression of *StBIN2* significantly enhanced salt tolerance in potato plants. Under salt stress conditions, the activities of CAT and SOD in the antioxidant system of transgenic *StBIN2* plants were elevated, while the contents of MDA and H_2_O_2_ were reduced. Collectively, these findings provide a theoretical reference for further understanding the potential role of *StBIN2* and its associated signaling pathways in potato adaptation to salt stress.

## Figures and Tables

**Table 1:** Information on Cis-acting elements in the *StBIN2* promoter region.

Cis-Acting Element	Sequence	Number	Element Function Description
WUN-motif	AAATTTCCCT	1	wound-responsive element
TGA-element	AACGAC	1	auxin-responsive element
TATC-box	TATCCCA	2	cis-acting element involved in gibberellin-responsiveness
Myb-binding site	CAACAG	1	
MYB recognition site	CCGTTG	1	
MYB like site	TAACCA	3	cis-acting element involved in salicylic acid responsiveness
TCA-elemwnt	CCATCTTTTT	1	
MYB	CAACAG	4	
W box	TTGACC	1	gibberellin-responsive element
P-box	CCTTTTG	1	part of a light responsive element
I-box	gGATAAGGTG	2	

## Data Availability

The data presented in this study are available within the article.

## Supplementary Materials

The following supporting information can be downloaded at: https://www.mdpi.com/article/10.3390/ijms27177747/s1.

## References

[1] Han X., Yang R., Zhang L., Wei Q., Zhang Y., Wang Y., Shi Y. (2023). A Review of Potato Salt Tolerance. Int. J. Mol. Sci..

[2] Akyol H., Riciputi Y., Capanoglu E., Caboni M.F., Verardo V. (2016). Phenolic Compounds in the Potato and Its Byproducts: An Overview. Int. J. Mol. Sci..

[3] McGill C.R., Kurilich A.C., Davignon J. (2013). The role of potatoes and potato components in cardiometabolic health: A review. Ann. Med..

[4] Valenzuela F.J., Reineke D., Leventini D., Chen C.C., Barrett-Lennard E.G., Colmer T.D., Dodd I.C., Shabala S., Brown P., Bazihizina N. (2022). Plant responses to heterogeneous salinity: Agronomic relevance and research priorities. Ann. Bot..

[5] Dasgupta S., Robinson E.J.Z. (2022). Attributing changes in food insecurity to a changing climate. Sci. Rep..

[6] Yang H., Chen Y., Zhang F. (2019). Evaluation of comprehensive improvement for mild and moderate soil salinization in arid zone. PLoS ONE.

[7] Tiwari R.K., Lal M.K., Kumar R. (2024). Salt stress influences the proliferation of Fusarium solani and enhances the severity of wilt disease in potato. Heliyon.

[8] Shen C., Yang W., Kang Y., Qin S., Zhang W., Liu Y., Qian S., Han Y. (2025). Effect of Alkaline Salt Stress on Photosynthetic Activities of Potato Plants (*Solanum tuberosum* L.). Plants.

[9] Nai G., Liang G., Ma W., Lu S., Li Y., Gou H., Guo L., Chen B., Mao J. (2022). Overexpression *VaPYL9* improves cold tolerance in tomato by regulating key genes in hormone signaling and antioxidant enzyme. BMC Plant Biol..

[10] Lv C., Bao Y., Xu M., Deng K., Zhao L., Zhao Y., Zhou Y., Feng Y., Wang F. (2025). Genome-Wide Identification of the *StPYL* Gene Family and Analysis of the Functional Role of StPYL9a-like in Salt Tolerance in Potato (*Solanum tuberosum* L.). Plants.

[11] Siddiqui M.H., Alamri S., Al-Khaishany M.Y., Khan M.N., Al-Amri A., Ali H.M., Alaraidh I.A., Alsahli A.A. (2019). Exogenous Melatonin Counteracts NaCl-Induced Damage by Regulating the Antioxidant System, Proline and Carbohydrates Metabolism in Tomato Seedlings. Int. J. Mol. Sci..

[12] Dong X., Sui C., Wang K., Chen W., Li Y., Kear P.J., Chang S., Lv D., Jian H. (2026). Melatonin enhances salt tolerance by inducing *StMYB55* to improve flavonoid accumulation in potato. Plant Physiol..

[13] Yuan P., Yang T., Poovaiah B.W. (2018). Calcium Signaling-Mediated Plant Response to Cold Stress. Int. J. Mol. Sci..

[14] Luo Y., Wang K., Zhu L., Zhang N., Si H. (2024). *StMAPKK5* Positively Regulates Response to Drought and Salt Stress in Potato. Int. J. Mol. Sci..

[15] Gruszka D. (2013). The brassinosteroid signaling pathway-new key players and interconnections with other signaling networks crucial for plant development and stress tolerance. Int. J. Mol. Sci..

[16] Li J., Zhou H., Zhang Y., Li Z., Yang Y., Guo Y. (2020). The GSK3-like Kinase BIN2 Is a Molecular Switch between the Salt Stress Response and Growth Recovery in *Arabidopsis thaliana*. Dev. Cell.

[17] Zhang K., Duan M., Zhang L., Li J., Shan L., Zheng L., Liu J. (2022). HOP1 and HOP2 are involved in salt tolerance by facilitating the brassinosteroid-related nucleo-cytoplasmic partitioning of the HSP90-BIN2 complex. Plant Cell Environ..

[18] Wang Y., Zhang G., Zhou H., Yin S., Li Y., Ma C., Chen P., Sun L., Hao F. (2023). GhPYL9-5D and GhPYR1-3 A positively regulate *Arabidopsis* and cotton responses to ABA, drought, high salinity and osmotic stress. BMC Plant Biol..

[19] Wang H., Tang J., Liu J., Hu J., Liu J., Chen Y., Cai Z., Wang X. (2018). Abscisic Acid Signaling Inhibits Brassinosteroid Signaling through Dampening the Dephosphorylation of BIN2 by ABI1 and ABI2. Mol. Plant.

[20] Wang Z., Li Z., Zhou X., Lu M., Ma Y., Zhang M., Liu Y., Gai Z., Yang K., Ren M. (2025). Saline-alkaline stress alters the drought resistance of maize through the ABA-PYL-SnRK2s signaling axis. Plant Physiol. Biochem..

[21] Cai Z., Liu J., Wang H., Yang C., Chen Y., Li Y., Pan S., Dong R., Tang G., Barajas-Lopez J.D. (2014). GSK3-like kinases positively modulate abscisic acid signaling through phosphorylating subgroup III SnRK2s in *Arabidopsis*. Proc. Natl. Acad. Sci. USA.

[22] Liu S., Cai C., Li L., Wen H., Liu J., Li L., Wang Q., Wang X. (2023). StSN2 interacts with the brassinosteroid signaling suppressor *StBIN2* to maintain tuber dormancy. Hortic. Res..

[23] Ye K., Li H., Ding Y., Shi Y., Song C., Gong Z., Yang S. (2019). Brassinosteroid-Insensitive2 Negatively Regulates the Stability of Transcription Factor ICE1 in Response to Cold Stress in *Arabidopsis*. Plant Cell.

[24] Jiang H., Tang B., Xie Z., Nolan T., Ye H., Song G.Y., Walley J., Yin Y. (2019). GSK3-like kinase BIN2 phosphorylates RD26 to potentiate drought signaling in *Arabidopsis*. Plant J..

[25] Zhang G., Hao C., Lou Y., Xi W., Wang X., Wang Y., Qu Z., Guo C., Chen Y., Zhang Y. (2010). Tissue-specific expression of TIPE2 provides insights into its function. Mol. Immunol..

[26] Matsumoto S., Kogure Y., Ono S., Numata T., Endo T. (2025). Msp1 and Pex19-Pex3 cooperate to achieve correct localization of Pex15 to peroxisomes. FEBS J..

[27] Wang H., Guan S., Zhu Z., Wang Y., Lu Y. (2013). A valid strategy for precise identifications of transcription factor binding sites in combinatorial regulation using bioinformatic and experimental approaches. Plant Methods.

[28] Singh A., Mehta S., Yadav S., Nagar G., Ghosh R., Roy A., Chakraborty A., Singh I.K. (2022). How to Cope with the Challenges of Environmental Stresses in the Era of Global Climate Change: An Update on ROS Stave off in Plants. Int. J. Mol. Sci..

[29] van Zelm E., Zhang Y., Testerink C. (2020). Salt Tolerance Mechanisms of Plants. Annu. Rev. Plant Biol..

[30] Zhou H., Shi H., Yang Y., Feng X., Chen X., Xiao F., Lin H., Guo Y. (2024). Insights into plant salt stress signaling and tolerance. J. Genet. Genomics.

[31] Song Y., Wang Y., Yu Q., Sun Y., Zhang J., Zhan J., Ren M. (2023). Regulatory network of GSK3-like kinases and their role in plant stress response. Front. Plant Sci..

[32] Luo J., Jiang J., Sun S., Wang X. (2022). Brassinosteroids promote thermotolerance through releasing BIN2-mediated phosphorylation and suppression of HsfA1 transcription factors in *Arabidopsis*. Plant Commun..

[33] Zhang J., Guo D., Zhang Y., Wei F., Han Q., Lao J., Wang H., Feng M., Luo X., Wang C. (2026). A BIN2-SOG1 molecular module regulates replication stress response independently on ATR. Sci. Adv..

[34] Kim H., Kang H., Kwon Y., Choi J., Chang J.H. (2019). Proportional subcellular localization of *Arabidopsis* thaliana RabA1a. Plant Signal. Behav..

[35] Zhang Q., Guo N., Zhang Y., Yu Y., Liu S. (2022). Genome-Wide Characterization and Expression Analysis of Pathogenesis-Related 1 (PR-1) Gene Family in Tea Plant (*Camellia sinensis* (L.) O. Kuntze) in Response to Blister-Blight Disease Stress. Int. J. Mol. Sci..

[36] Lei Y.Y., Ma Z., Wei J., Li W.B., Li Y., Yang Z.M., Zhang S.S., Feng J.Q., Sheng H.C., Liu Y. (2025). Identification of GSK3 family and regulatory effects of brassinolide on growth and development of Nardostachys jatamansi. China J. Chin. Mater. Med..

[37] Sun F., Deng Y., Zhao W., Xiong Y., Zhao L., Zhang L. (2025). High-throughput quantitative assessment of ABA-responsive elements at single-nucleotide resolution. Quant. Biol..

[38] Mena M., Cejudo F.J., Isabel-Lamoneda I., Carbonero P. (2002). A role for the DOF transcription factor BPBF in the regulation of gibberellin-responsive genes in barley aleurone. Plant Physiol..

[39] Sugihara F., Kasahara K., Kokubo T. (2011). Highly redundant function of multiple AT-rich sequences as core promoter elements in the TATA-less RPS5 promoter of Saccharomyces cerevisiae. Nucleic Acids Res..

[40] Dai X., Xu Y., Ma Q., Xu W., Wang T., Xue Y., Chong K. (2007). Overexpression of an *R1R2R3* MYB gene, *OsMYB3R-2*, increases tolerance to freezing, drought, and salt stress in transgenic *Arabidopsis*. Plant Physiol..

[41] Khoza T., Masenya A., Khanyile N., Thosago S. (2025). Alleviating Plant Density and Salinity Stress in Moringa oleifera Using Arbuscular Mycorrhizal Fungi: A Review. J. Fungi.

[42] Wang P., Du Y., Li Y., Ren D., Song C.P. (2010). Hydrogen peroxide-mediated activation of MAP kinase 6 modulates nitric oxide biosynthesis and signal transduction in *Arabidopsis*. Plant Cell.

[43] Rohman M.M., Islam M.R., Monsur M.B., Amiruzzaman M., Fujita M., Hasanuzzaman M. (2019). Trehalose Protects Maize Plants from Salt Stress and Phosphorus Deficiency. Plants.

[44] Tsikas D. (2017). Assessment of lipid peroxidation by measuring malondialdehyde (MDA) and relatives in biological samples: Analytical and biological challenges. Anal. Biochem..

[45] Guo D.F., Uno S., Ishihata A., Nakamura N., Inagami T. (1995). Identification of a cis-acting glucocorticoid responsive element in the rat angiotensin II type 1A promoter. Circ. Res..

[46] Liu S., Cai C., Li L., Yu L., Wang Q., Wang X. (2024). Transcriptome Analysis Reveals the Molecular Mechanisms of BR Negative Regulatory Factor *StBIN2* Maintaining Tuber Dormancy. Int. J. Mol. Sci..

[47] Lye C.M., Naylor H.W., Sanson B. (2014). Subcellular localisations of the CPTI collection of YFP-tagged proteins in Drosophila embryos. Development.

[48] Rao X., Huang X., Zhou Z., Lin X. (2013). An improvement of the 2ˆ(−delta delta CT) method for quantitative real-time polymerase chain reaction data analysis. Biostat. Bioinforma. Biomath..

[49] Awasthi B.P., Guragain D., Chaudhary P., Jee J.G., Kim J.A., Jeong B.S. (2023). Antitumor activity of a pexidartinib bioisostere inhibiting CSF1 production and CSF1R kinase activity in human hepatocellular carcinoma. Chem. Biol. Interact..

[50] Bittner N.K.J., Mack K.L., Nachman M.W. (2022). Shared Patterns of Gene Expression and Protein Evolution Associated with Adaptation to Desert Environments in Rodents. Genome Biol. Evol..

[51] Liu J., Cai C., Liu S., Li L., Wang Q., Wang X. (2023). *StBIN2* Positively Regulates Potato Formation through Hormone and Sugar Signaling. Int. J. Mol. Sci..

