# Supplementary material for: Overexpression of StBIN2 Improves Salt Stress Tolerance in Potato
Source: Int J Mol Sci. 2026 Aug 29;27(17):7747. doi: 10.3390/ijms27177747 (PMC13566549; doi:10.3390/ijms27177747)
Supplement: Supplementary file 1 [file ijms-27-07747-s001.zip › ijms-4487107-supplementary/Supplementary S1.pdf]

The appendix is Arabidopsis GSK3 family protein sequence

>At5G26750

MASVGIAPNPGARDSTGVDKLPEEMNDMKIRDDKEMEATVVDGNGTETGHIIVTTIGGR
NGQPKQTISYMAERVVGHGSFGVVFQAKCLETGETVAIKKVLQDRRYKNRELQTMRLLD
HPNVVSLKHCFFSTTEKDELYLNLVLEYVPETVHRVIKHYNKLNQRMPLIYVKLYTYQIFR
ALSYIHRCIGVCHRDIKPQNLLVNPHTHQVKLCDFGSAKVLVKGEPNISYICSRYYRAPELI
FGATEYTTAIDVWSAGCVLAELLLGQPLFPGESGVDQLVHIIKVLGTPTREEIKCMNPNYT
EFKFPQIKAHPWHKIFHKRMPPEAVDLVSRLLQYSPNLRSAALDTLVHPFFDELRDPNARL
PNGRFLPPAFHFKPHELKGVPLEMVAKLVPEHARKQCPWLGL

>At3g05840

MASVGIEPSAAVRESTGNVTDADRLPEEMKDMKIQDDKEMEATIVNGNVTETGHIIVTTI
GGRNGQPKQTISYMAERVVGHGSFGVVFQAKCLETGETVAIKKVLQDRRYKNRELQTMR
LLDHPNVVSLKHCFFSTTEKDELYLNLVLEYVPETVHRVIKHYNKLNQRMPLVYVKLYTY
QIFRSLSYIHRCIGVCHRDIKPQNLLVNPHTHQVKLCDFGSAKVLVKGEPNISYICSRYYRA
PELIFGATEYTTAIDVWSAGCVLAELLLGQPLFPGESGVDQLVEIIKVLGTPTREEIKCMNP
NYTEFKFPQIKAHPWHKIFHKRMPPEAVDLVSRLLQYSPNLRCAALDSLVHPFFDELRDPN
ARLPNGRFLPPLFNFKPHELKGVPVEMVAKLVPEHARKQCPWLSL

>At5g14640

MASVGTLPASSMATKQSNASICAEKLPEGINEMKIKDDKEMEAAVVDGNGTETGHIIVTTI
GGKNGQPKQTISYMAERIVGQGSFGIVFQAKCLETGETVAIKKVLQDKRYKNRELQTMRL
LDHPNVVSLKHCFFSTTEKDELYLNLVLEYVPETVYRVSKHYSRANQRMPIIYVKLYTYQI
CRALAYIHGGVGVCHRDIKPQNLLVNPHTHQVKLCDFGSAKVLVKGEPNISYICSRYYRA
PELIFGATEYTTTIDIWSAGCVLAELLLGQPLFPGESGVDQLVEIIKVLGTPTREEIKCMNPN
YTEFKFPQIKAHPWHKIFHKRTPPEAVDLVSRLLQYSPNLRSTAMEAIVHPFFDELRDPNTR
LPNGRALPPLFNFKPQELKGASLELLSKLIPDHARKQCSFLAL

>At1G06390

MASLPLGPQPHALAPPLQLHDGDALKRRPELDSDKEMSAAVIEGNDAVTGHIISTTIGGKN
GEPKQTISYMAERVVGTGSFGIVFQAKCLETGESVAIKKVLQDRRYKNRELQLMRPMDHP
NVISLKHCFFSTTSRDELFLNLVMEYVPETLYRVLRHYTSSNQRMPIFYVKLYTYQIFRGLA
YIHTVPGVCHRDVKPQNLLVDPLTHQVKLCDFGSAKVLVKGEPNISYICSRYYRAPELIFG
ATEYTASIDIWSAGCVLAELLLGQPLFPGENSVDQLVEIIKVLGTPTREEIRCMNPNYTDFR
FPQIKAHPWHKVFHKRMPPEAIDLASRLLQYSPSLRCTALEACAHPFFNELREPNARLPNG
RPLPPLFNFKQELGGASMELINRLIPEHVRRQMSTGLQNS

>At2g30980

MTSIPLGPPQPPSLAPQPPHLHGGDSLKRRPDIDNDKEMSAAVIEGNDAVTGHIISTTIGGK
NGEPKQTISYMAERVVGTGSFGIVFQAKCLETGESVAIKKVLQDRRYKNRELQLMRLMD
HPNVVSLKHCFFSTTTRDELFLNLVMEYVPETLYRVLKHYTSSNQRMPIFYVKLYTYQIFR
GLAYIHTAPGVCHRDVKPQNLLVDPLTHQCKLCDFGSAKVLVKGEANISYICSRYYRAPEL
IFGATEYTSSIDIWSAGCVLAELLLGQPLFPGENSVDQLVEIIKVLGTPTREEIRCMNPNYTD
FRFPQIKAHPWHKVFHKRMPPEAIDLASRLLQYSPSLRCTALEACAHPFFNELREPNARLP
NGRPLPPLFNFKQELSGASPELINRLIPEHVRRQMNGGFPFQAGP

>At4g18710

MADDKEMPAAVVDGHDQVTGHIISTTIGGKNGEPKQTISYMAERVVGTGSFGIVFQAKCL
ETGETVAIKKVLQDRRYKNRELQLMRVMDHPNVVCLKHCFFSTTSKDELFLNLVMEYV

PE SLYRVLKHYSSANQRMPLVYVKLYMYQIFRGLAYIHNVAGVCHRDLKPQNLLVDPLT
HQVKICDFGSAKQLVKGEANISYICSRFYRAPELIFGATEYTTSIDIWSAGCVLAELLLGQP
LFPGENAVDQLVEIIKVLGTPTREEIRCMNPHYTDFRFPQIKAHPWHKIFHKRMPPEAIDFA
SRLLQYSPSLRCTALEACAHPFFDELREPNARLPNGRPFPPLFNFKQEVAGSSPELVNKLIP
DHIKRQLGLSFLNQSGT

>At3G61160

MNVVRRLTSIASGRNFVSSDNVGETETPRSKPNQNREETESTETTSYEKDSVSSSENSDHL
PKEIREVGLGDDKDMDCGIIKGNGTESGRIITTKKKGLNDQKDKTISYRAEHVIGTGSFGV
VFQAKCLETEEKVAIKKVLQDKRYKNRELQIMRMLDHPNVVELKHSFFSTTEKDELYLNL
VLEYVPETIYRASRSYTKMNQHMPLIYIQLYTYQICRAMNYLHQVVGVCHRDIKPQNLLV
NNVTHEVKICDFGSAKMLIPGEPNISYICSRYYRAPELIFGATEYTSAIDMWSVGCVMAEL
FLGHPLFPGETSVDQLVEIIKILGTPAREEIKNMNPRYNDFKFPQIKAQPWHKIFRRQVSPEA
MDLASRLLQYSPNLRCTALEACAHPFFDDLRDPRASLPNGRALPPLFDFTAQELAGASVE
LRHRLIPEHAR

>At4G00720

MNVMRRLKSIASGRTSISSDPGGDYALKRAKLDQENDNLCVDPMQVDQNSSCFEMKAD
VLSQESVAGTSNVPAVSEKPVDDQLPDVMIEMKIRDERNANREDKDMETTVVNGSGTET
GQVITTTVGGRDGKPKQTISYMAQRVVGTGSFGVVFQAKCLETGEQVAIKKVLQDKRYK
NRELQIMRLQDHPNVVRLRHSFFSTTDKDELYLNLVLEYVPETVYRASKHYTKMNQHMP
IIFVQLYTYQICRALNYLHRVVGVCHRDIKPQNLLVNPQTHQLKICDFGSAKMLVPGEPNIS
YISRYYRAPELIFGATEYTNAIDMWSGGCVMAELLLGQPLFPGESGIDQLVEIIKILGTPTRE
EIRCMNPNYTEFKFPQIKAHPWHKIFHKRMPPEAVDLVSRLLQYSPNLRCTALEACAHPFF
DDLRDPNVSLPNGRALPPLFNFTAQELAGASTELRQRLIPAHCQGTGSSS

>At1G09840

MASSGLGNGVGTSRSAKGLKSSSSSVDWLTRDLAETRIRDKVETDDERDSEPDIIDGAGA
EPGHVIRTTLRGRNGQSRQTVSYISEHVVGTGSFGMVFQAKCRETGEVVAIKKVLQDKRY
KNRELQIMQMLDHPNAVALKHSFFSRTDNEEVYLNLVLEFVPETVNRVARSYSRTNQLM
P LIYVKLYTYQICRALAYIHNSFGLCHRDIKPQNLLVNPHTHQLKICDFGSAKVLVKGEPN
VSYICSRYYRAPELIFGASEYTTAIDIWSTGCVMAELLLGQPLFPGESGVDQLVEIIKVLGTP
TREEIKCMNPNYTEFKFPQIKPHPWHKVFQKRLPPEAVDLLCRFFQYSPNLRCTALEACIH
PLFDELRDPNTRLPNGRPLPPLFNFKPQELSGIPPEIVNRLVPEHARKQNLFMALHS

>At1G57870

MESHLGNGVGSSRSAKNTKNTSSSVDWLSRDMLEMKIRDKTEADEERDSEPDIIDGVGA
EPGHVITTTLPGRNGQSRQTVSYIAEHVVGTGSFGMVFQAKCRETGEVVAIKKVLQDKRY
KNRELQIMQMLDHPNVVCLKHSFYSRTENEEVYLNLVLEFVPETVNRTARSYSRMNQLM
PLIYVKLYTYQICRGLAYLHNCCGLCHRDIKPQNLLVNPHTHQLKICDFGSAKVLVKGEPN
ISYICSRYYRAPELIFGATEYTTAIDIWSTGCVMAELLLGQPLFPGESGVDQLVEIIKVLGTP
TREEIKCMNPNYTEFKFPQIKPHPWHKVFQKRLPPEAVDLLCRFFQYSPNLRCTAVEACIH
PFFDELRDPNARLPNGRPLPPLFNFKPQELSGIPPETVDRLVPEHARKQNHFMALHSCLRIP
CREVRMRKIQHYFVRYMI
