# Supplementary material for: Overexpression of StBIN2 Improves Salt Stress Tolerance in Potato
Source: Int J Mol Sci. 2026 Aug 29;27(17):7747. doi: 10.3390/ijms27177747 (PMC13566549; doi:10.3390/ijms27177747)
Supplement: Supplementary file 1 [file ijms-27-07747-s001.zip › ijms-4487107-supplementary/Supplementary table S1.pdf]

Gene name	Sequence
qStBIN2	GTTGTTTTTCAGGCAAAATG
	ATACTTGATACGTGTAAAGC
